# An epidemiological study on face masks and acne in a Nigerian population

**DOI:** 10.1371/journal.pone.0268224

**Published:** 2022-05-19

**Authors:** Olanrewaju Falodun, Nubwa Medugu, Laila Sabir, Ihsan Jibril, Nnebuogo Oyakhire, Adeola Adekeye

**Affiliations:** 1 Dermatology Unit Department of Internal Medicine, National Hospital Abuja, Abuja, Nigeria; 2 Department of Medical Microbiology and Parasitology, National Hospital Abuja, Abuja, Nigeria; Freelance Consultant, Myanmar, MYANMAR

## Abstract

**Background:**

Acne vulgaris is a skin disorder that affects males and females with significant impact on quality of life. The onset of the COVID-19 pandemic led to a series of non-pharmaceutical interventions globally to reduce the spread of the virus particularly since there have been no known cures or definitive treatment for the disease. One key non-pharmaceutical intervention was recommendation on wearing of face masks. There are reports of discomfort associated with wearing face mask including complaints of various skin rashes, acne and headaches which could hinder appropriate use of face masks. While the dermatological problems associated with face mask use have been comprehensively explored in high income countries, the data is sparse in sub-Saharan Africa. We aimed to determine the association between face mask use and development of acne vulgaris in our developing country setting. We subsequently determined risk factors for development of acne vulgaris such as duration of wearing face masks, type of face mask, and prior dermatological skin condition history. We aimed to also determine the potential of acne development secondary to face mask use to reducing predisposition to wearing face masks.

**Methods:**

This was an observational cross-sectional study conducted in within two local government areas of the Federal Capital Territory, Abuja. Trained interviewers administered pre-tested questionnaires to 1316 consecutive consenting adult participants randomly approached for informed consent at various public locations. Information was inputted into MS Excel and analyzed using Epi-info.

**Results:**

A total number of 1316 persons participated in this study with mean age 34.4 ±12.3 years and median age 35.5years. Male: female ratio was 1:1.41. New onset acne or worsening of acne following consistent wearing of face masks was reported by 323 (24.5%) of the 1316 participants in this study. The surgical face mask was the least likely to predispose to acne p<0.05. Compared with the surgical mask, persons using N95 face mask and cloth mask were 1.89 and 1.41 times more likely to have acne respectively. Persons with prior history of acne were more likely to develop new acne or experience worsening of acne following wearing of face mask OR 3.89, 95% CI 2.85, 5.33; p <0.05). The length of time of daily mask wearing was not significantly associated with occurrence of new onset acne or worsening of acne. Persons reporting prior histories of allergy were more likely to develop acne in this study (OR 2.01, 95% CI 1.50, 2.88; p<0.05). In this study, 192 (59.4%) of those who reported having acne following face masks use responded they have a negative predisposition to wearing masks.

**Conclusion:**

Our finding of greater predisposition to development or worsening of acne following consistent use of face masks could have implications for the control strategy of COVID-19. The finding that the N95 face mask was more significantly associated with acne is of concern as this is the preferred face mask in healthcare settings. It is important for the medical community to investigate feasible and safe recommendations to help alleviate this condition.

## Introduction

The COVID-19 respiratory disease caused by the SARS-CoV-2 virus has caused significant morbidity and mortality worldwide [1, 2]. Because the disease spreads primarily via the respiratory route, measures limiting spread of respiratory droplets and aerosols amongst people were implemented to reduce the rate of transmission of the virus. The measures were generally non pharmaceutical interventions (NPIs) such as physical distancing and wearing of face masks. Several studies have shown that NPIs such as the wearing of face mask have resulted in significant reduction in the projected magnitude of the outbreak [3, 4]. The development and deployment of COVID-19 vaccinations brought a ray of hope that face masks would be phased out as a primary strategy for COVID-19 control, there was however global continued mandatory regulations on wearing of face masks particularly since vaccine coverage–especially in low-middle income countries (LMICs) continues to be low [5, 6]. Face masks also remain a vital preventive component of COVID-19 as vaccines may not be equally protective across emerging strains of COVID-19 [7, 8].

While the importance of face mask use in preventing transmission of COVID-19 cannot be over emphasized, it does come with attendant dermatological problems such as rosacea, dermatitis, and acne as reported in high income countries [9, 10]. Acne may result in severe discomfort, itching, pain, with superimposed bacterial infection. Acne may also lead to refusal to wear face masks and consequent continued spread of respiratory pathogens. Acne vulgaris is a dermatological condition arising from interaction of numerous factors which eventually result in the formation of lesions of acne on the face and parts of the body. These factors include hyperkeratosis of sebaceous follicles, stimulating effect of androgens on the sebaceous glands and the effect of *Propionibacterium acne* which contribute to inflammation culminating in acne vulgaris [11]. The term “Maskne “refers to acne breakouts along the jaw, cheeks and mouth which are areas covered by face masks hence the general inference that maskne is triggered by wearing of face masks [9]. Some authors have linked the occurrence of maskne to the effects of microbiome dysbiosis resulting from factors such as increased humidity and bacteria trapping within the mask space, friction between parts of mask touching facial skin, and temperature changes due to prolonged wearing of masks [12–14]. Studies in developed countries have found the different mask types to be variably associated with maskne [15–17] although the potential association has not been described in sub-Saharan Africa.

Here, we aimed to determine associations between face masks and acne among the study population. We also aimed to determine if the type of face mask worn and duration of wearing face masks would be associated with the outcomes of interest. Lastly, we aimed to determine if the occurrence or worsening of acne adversely affected predisposition of study participants to wearing face masks.

## Materials and methods

This study was a cross sectional study using a cluster sampling method carried out from 1st July 2021 to 25th October 2021. Ethical approval (No EC/055/2021) was obtained from the National Hospital Ethics Research Committee and verbal informed consent was obtained from all participants. Two local government areas within the Federal capital Territory i.e, Abuja Municipal Area Council and Gwagwalada Area Council were selected by simple random sampling out of the six local Government areas in the Federal Capital Territory. The population estimates for the two randomly selected area councils were 1,967,500 for Abuja Municipal Area Council and 402,000 for Gwagwalada Area council [18].

Participants were consecutive consenting volunteers aged at least 18 years and residing in any of the two local government areas. Study recruited 1316 participants, of which 1096 were recruited in Abuja Municipal Area council, while 220 participants were recruited in Gwagwalada Area Council based on their proportionate populations. The interviewers administered the questionnaires to random people attending religious centers, markets, shopping malls, schools, hospitals, and bus stops. These interviewers were National Youth Corp members posted to National Hospital Abuja for the Nigerian one-year mandatory service year and offered themselves as volunteers for the study. These corps members were trained before the pilot testing of the questionnaire and participated in the pilot testing before being deployed to field. On approaching potential participants, the corps members introduced themselves, stated the purpose of the study and enquired if the person would like to participate by answering questions for 5–8 minutes. Verbal informed consent was then obtained. Participants younger than 18 years and persons not willing to participate were excluded from the interviews. The demographic information included in the questionnaire were age, gender, and occupation. Other questions administered to the participants included type of masks worn most frequently, duration of wearing of masks, previous or current history of allergies and acne. The questionnaires were pre-tested among random people in the community before full deployment for the study. Identifiers such as names and phone numbers were not collected for the study and hence confidentiality was maintained through the course of data collection. All questionnaires were printed as hard copies and used as such by the interviewers to administer to participants. The hard copies were transcribed unto Microsoft Excel by the research assistants and double checked by the study coordinator.

The excel sheet generated was used as data source input for EPI INFO^(R)^ version 7.2.4 which was used for data analysis. Demographic data were analyzed and expressed as means, median, frequencies and standard deviations. Categorical variables were compared using Chi-square test and reported by proportions. Univariate and multivariate analysis were performed to test associations between proposed factors and onset of acne or worsening of acne vulgaris. Factors biologically plausible as being related to development of acne were added in the multivariate regression model. Results of analysis were presented in tables. Statistical significance was indicated at a p value of < 0.05 with 95% confidence interval.

## Results

A total of 1573 persons were approached to participate in the study out of which 1316 persons responded in this study (83.7% response rate). The respondents consisted of 58.6% females (n = 771) and 41.4% males (n = 545). The 21–30-year age group were the most frequent age group represented in the sample population. Nearly 20% of the participants were healthcare workers.

The age of participants ranged from 18 years to 56 years. The mean age of participants is 34.4 ±12.3 years while the median age was 35.5 years. The demographic details are shown in more detail in Table 1 below.

**Table 1:** Sociodemographic data of participants.

Variable	Number (n = 1316)	%
**Age range**		
18–20	238	18.1
21–30	333	25.3
31–40	322	24.5
41–50	252	19.2
51–56	171	13.0
**Gender**		
Female	771	58.6
Male	545	41.4
**Occupation**		
Health worker	263	19.9
Business	245	18.6
Self- employed	213	16.2
Unemployed	184	14.0
Civil servant	139	10.6
Farmer	135	10.3
Student	118	9.0
Other	19	1.4

New onset acne or worsening of acne was reported by 323 (24.5%) of the 1316 participants in this study. Of this number 58 (18%) developed acne for the first time following wearing of face mask. This number represented 4.4% of the 1316 study participants. Participants with prior history of acne were 3.9 times more likely to report new or worsening acne following increased use of face mask compared with those without such prior history. The type of mask used was significantly associated with development of new or worsening acne with the most likely to develop acne being those participants who use N95 face mask followed by those using cloth mask. The surgical face mask was the least likely to predispose to acne p<0.05. Compared with the surgical mask, persons using N95 face mask and cloth mask were 1.89 and 1.41 times more likely to have acne respectively.

Among those who reported development of new onset acne or worsening of previous acne following consistent use of face mask, 192 of them (59.4%) reported that they were less inclined to wear face masks because of the condition.

## Discussion

In this study, we observed that about a quarter of the population reported new onset acne or worsening of preexisting acne. The NPIs implemented to control the spread of COVID-19 have proven to be very useful in flattening the curve of the pandemic which has caused unprecedented devastation globally [3, 4]. Wearing of face masks is one of the key components of the NPIs but this appears to have its attendant consequences [14, 19]. In this study, we observed that about a quarter of the population reported new onset acne or worsening of preexisting acne. The 24.5% of the population sampled reporting acne is lower than that reported in studies in Singapore and Pakistan although these studies focused on healthcare workers who are thus more likely to wear face masks more consistently and for longer periods of time [20, 21]. These numbers are high enough to be of concern especially as wearing of face masks is a key aspect of global NPI to reduce the spread of COVID-19.

We found no sex predilection in occurrence of acne in this study. This is inconsistent with findings by other studies in Turkey and Thailand [15, 19] where females were reported as more likely to develop maskne. Our finding that persons with prior history of acne vulgaris were about four times more likely to develop new acne lesions or experience worsening of their acne (Table 2) is logical and may be linked to their background predisposition to having acne prior to wearing face masks.

**Table 2:** Risk factors to development of new or worsening acne.

	New/worsening acne n (%) N = 323	No New/worsening acne n (%) N = 966	P value	aOR (95% CI)
**Gender**				
**Female**	181 (56.0)	576 (59.6)	0.27	0.86 (0.67, 1.11)
**Male**	142 (44.0)	390 (40.1)		
**Prior history of acne**				
**Yes**	265 (82.0)	522 (54.0)	<0.05	3.89 (2.85, 5.33)
**No**	58 (18.0)	444 (46.0)		
**Prior history of allergies**				
**Yes**	72 (22.9)	117 (12.1)	<0.05	2.01 (1.50, 2.88)
**No**	251(77.7)	849 (87.9)		
**Type of mask used**				
**Cloth mask/Surgical mask**	119 (28.17)	304 (71.9)	0.01	1.41(1.07, 1.86)
**N95/Surgical mask**	44 (13.6228.4)	84 (65.6)	<0.05	1.89 (1.26,2.84)
**Surgical mask**	160 (21.68)	578 (78.3)	<0.05	*
**Duration of hours with mask daily**				
**1–2 hrs.**	75 (25.0)	225 (74.0)		
**3–4 hrs.**	91 (24.4)	282 (75.6)	0.85	0.97 (0.68,1.38)
**5–8 hrs.**	80 (21.7)	289 (78.3)	0.31	0.83 (0.58, 1.19)
**9–12 hrs.**	58 (30.7)	131 (69.3)	0.97	1.33 (0.89, 2.00)
**>12 hrs.**	19 (32.8)	39 (67.2)	0.22	1.46 (0.79, 2.68)

Our finding of increased likelihood of acne development during consistent use of face masks by persons with previous acne history has been reported elsewhere [13, 22]. The relatively high ambient temperatures in tropical regions like Nigeria result in more sweating beneath the mask and a consequent rise in the humidity of surrounding skin which causes swelling of keratinocytes of the pilosebaceous follicles. The resultant acute obstruction can lead to acne aggravation [23]. The higher temperatures and increased humidity within the face mask during use could lead to increased sebum excretion and optimal growth conditions for bacteria associated with acne such as *Propionibacterium acne*. This hypothesis is supported by similar studies across other countries [21, 24]. In this study, we found that persons with prior history of allergies were more than twice as likely to develop acne as those without history of allergies. While a face mask induced itch has been described by Jacek et al. [25], there is currently no documented causal relationship between acne and allergies–hence a novel finding which should be investigated further. We hypothesize here that this association could be because of the common inflammatory pathway shared by both conditions.

While the N95 face mask is thought to be most protective against diseases transmitted via the airborne or droplet route, it appears the least protective against acne as found in our study and also reported in other studies [15, 26]. We postulate that this may result from inappropriate re-use of this mask. It is commonplace in low resource settings such as ours to re-use these face masks many times–sometimes up to ten times. The sweating and inadvertent spread of skin microbiota to the mask may create good growth conditions for the microbes which subsequently recolonize the skin in greater numbers when the mask is worn subsequently. A report by Tan KT and Greaves MN in 2004 observed in a Singapore General Hospital that two hospital staff who wore N95 masks continually for 3 months while working on the wards during the severe acute respiratory distress (SARS) crisis subsequently developed nodular acne lesions around the areas covered by the masks [27]. These findings further emphasize the need for rational use of personal protective equipment. We recommend that the regular surgical mask be the rational PPE to use in low resource settings because it still offers a measure of protection against respiratory microorganisms and acne rather than N95 masks that are worn, torn, wet, and have been reused or recycled too often. Regulatory authorities should ensure rational use of the N95 face mask to ensure it is used where most needed–in health care facilities.

We do recommend in the context of our finding for more discretion on types of masks used among those with history of allergies or ongoing allergies to decrease acne breakouts. We recommend that appropriate allergy management and adhering to dermatologist skin care guidance be implemented as part of acne prevention measures among face mask users.

Individuals with known allergies especially to fabrics also need to be selective about the cloth masks they wear daily outside the hospital environment. In this study, we did not observe an association between duration of wearing masks and occurrence of acne. This is in contrast with other studies where longer duration of wearing masks was associated with higher incidence of acne [19, 28, 29]. There was a large proportion of healthcare workers among the respondents in this study. This may be partially attributable to more knowledge about the importance in participating in research or the willingness to help the younger ‘healthcare’ workers fulfill their duties. It was concerning to discover that in this study population, 59.4% of those with maskne had a negative predisposition to wearing face masks. This is an urgent call to investigate preventive and curative modalities for acne among consistent users of face masks. To trivialize the issue will only mean that as time goes on many people particularly health care workers who are required to wear face masks for long periods including N95 masks may become demotivated and may expose themselves to risks while trying to avoid acne vulgaris. Dermatologists and other health workers need to discuss more about this condition and educate the populace around them on how to prevent and treat mask induced acne “Maskne” particularly as it seems the practice of wearing face masks may remain with us for a while longer than imagined previously.

## Conclusion

The findings from this study show that mask induced acne is a significant problem that requires more attention and focus. This is crucial because the occurrence of acne can hinder the individuals from wearing masks. It is important for the medical community to investigate feasible and safe recommendations to help alleviate this condition. The N95 mask used by health workers has the greatest tendency to causing mask induced acne and its use should only be as indicated after risk assessment.

### Limitations of the study

One primary limitation was that this study relied on the participant’s self-reporting of acne which in some cases may not be accurate. The recall of previous history of allergies was non-specific and could also been prone to recall bias. Another limitation was absence of a clinical component to this study where a dermatologist confirmation of the diagnosis would have been more objective.

## Supporting information

S1 Checklist STROBE (Strengthening The Reporting of OBservational studies in Epidemiology) checklist. (PDF)

S1 Data (XLSX)

S1 File Epidemiological study on face masks and acne in a Nigerian population. (DOCX)
