# Supplementary material for: An epidemiological study on face masks and acne in a Nigerian population
Source: PLoS One. 2022 May 19;17(5):e0268224. doi: 10.1371/journal.pone.0268224 (PMC9119463; doi:10.1371/journal.pone.0268224)
Supplement: S1 File — (DOCX) [file pone.0268224.s003.docx]

**EPIDEMIOLOGICAL STUDY ON FACE MASKS AND ACNE IN A NIGERIAN POPULATION**

Dear respondent I am a Medical Doctor working in a hospital in Abuja and I am carrying out research to find out possible associations between wearing of face masks and acne. This study intends to add more knowledge on the subject with a view to further finding solutions in future studies.

I will be grateful if you can help respond to this questions underlisted. You however may wish to decline without any consequences whatsoever.

By agreeing to fill this form it is taken that you have given your consent to participate in this study. Thank you.

**Demographic Details No………….**

(1) Age ……………………………

(2) Sex (i) Male (ii)Female

(3) Occupation (i) self-employed (ii) farming (iii) health worker (iv) Civil servant other than health worker (v) unemployed

**Medical History**

(4) Do you have any known allergies? I) Yes (ii) No

(5) If yes to no 4 what allergies please? ……………………………

**Use of face masks.**

(6) For how long do you wear face masks each day? (i) 2hours (ii)2-4hours (iii) 4-8hours (iv) 8-12hours (v)above 12hours

(7) Which type do you wear most often (i) cloth/fabric (ii) Surgical masks (iii) N95

(8) Did you have acne before COVID -19 pandemic (i) Yes (ii) No

(9) If yes to no 8, was it mild, moderate, or severe by your assessment………………………….

(10) Have you noticed worsening of acne (i) Yes (ii) No

(11) If no to question 8. Have you developed acne after regular use of face masks? (i)Yes (ii) No

(12) Has the occurrence of acne affected your use of face masks negatively (i) Yes (ii) No

(13) How long did the acne last? (i) < 1month (ii) 1month -3months (iii) 3months -6months (iv) >6months

(14) Did you seek medical attention for the acne (i) Yes (ii)No

Thanks for answering these questions.
